# Diabetes Prevalence and Associated Factors Among Pasifika Adults in Australia: Findings from the Pasifika Preventing Diabetes Programme

**DOI:** 10.3390/medsci14030384

**Published:** 2026-07-09

**Authors:** Kegnie Shitu, David Simmons, Kate McBride, Greg Gamble, Valentina Razmovski-Naumovski, Ronda Thompson, Timothy Low-Wah, Siaosi Tene Tiueti Pahulu, Makeleta Felila, Wadad Kathy Tannous, Salima Olataga Alofivae-Doorbinnia, Emily Hibbert, Ngai Wah Cheung, Mandy Williams, Freya MacMillan

**Affiliations:** 1 School of Health Sciences, Faculty of Health, Western Sydney University, Campbelltown, NSW 2560, Australia; k.getie@westernsydney.edu.au; 2 College of Medicine and Health Sciences, University of Gondar, Gondar P.O. Box 196, Ethiopia; 3 School of Medicine, Faculty of Health, Western Sydney University, Campbelltown, NSW 2560, Australia; 4 Translational Health Research Institute, Western Sydney University, Campbelltown, NSW 2560, Australia; 5 Faculty of Medical and Health Sciences, University of Auckland, Auckland 1023, New Zealand; 6 Health Promotion, South Western Sydney Local Health District, Campbelltown, NSW 2560, Australia; 7 Health Promotion, Western Sydney Local Health District, North Parramatta, NSW 2150, Australia; 8 School of Business, Translational Health Research Institute, Parramatta, NSW 2150, Australia; 9 Powell St Family Medical Practice, Yagoona, NSW 2199, Australia; 10 Faculty of Medicine and Health, The University of Sydney, Camperdown, NSW 2050, Australia; 11 Department of Endocrinology, Nepean Hospital, Kingswood, NSW 2747, Australia; 12 Department of Diabetes & Endocrinology, Westmead Hospital, Westmead, NSW 2145, Australia; 13 Population Health, South Western Sydney Local Health District, Liverpool, NSW 2170, Australia; 14 Division of Research and Innovation, Western Sydney University, Penrith, NSW 2751, Australia

**Keywords:** community-based, diabetes mellitus, HbA1c, obesity, Pasifika, physical activity, prevalence

## Abstract

**Background:** Given the paucity of evidence on diabetes and associated risk factors among Pasifika communities in Australia, this analysis aimed to estimate the prevalence of diabetes and associated risk factors in this population. **Methods:** A whole-of-community-based cross-sectional analysis was conducted using baseline health screening data from adults aged ≥18 years in the Pasifika Preventing Diabetes Programme, a stepped-wedge randomised controlled trial evaluating the effectiveness of a “through the church” behaviour change intervention on diabetes prevention and management in Pasifika communities in Greater Sydney. HbA1c, random blood glucose, blood pressure, and anthropometric measurements were collected alongside sociodemographic, health behaviour, diabetes knowledge, and quality-of-life questionnaire data. Diabetes was defined by HbA1c ≥ 6.5% or self-reported diagnosis. Multivariable binary logistic regression was conducted to identify significant factors associated with the odds of having diabetes. **Results:** Among 1161 participants, 33.9% (95% CI: 31.1–36.7) had diabetes, of whom 34.3% were previously undiagnosed. Only 2.5% met fruit and vegetable guidelines; 34.3% met minimum physical activity recommendations, 95.8% were obese/overweight, and 68.0% had high blood pressure. The adjusted odds ratio (AOR) of having diabetes increased with family history of diabetes (AOR, 2.23; 95% CI: 1.56–3.20), high blood pressure (AOR, 1.65; 95% CI: 1.15–2.38), and older age (AOR, 1.06; 95% CI: 1.05–1.07). A high level of physical activity was associated with a 41% lower odds of diabetes compared to a low level of physical activity (AOR, 0.59; 95% CI: 0.38–0.94). **Conclusions:** This study demonstrates a high burden of diabetes among Pasifika adults in Australia, including a substantial proportion of previously undiagnosed cases. Older age, family history of diabetes, and high blood pressure were associated with higher odds of diabetes, while high physical activity was associated with lower odds. These findings highlight the need for culturally safe, community-based approaches to strengthen diabetes screening, early diagnosis, and integrated prevention strategies in this underserved high-risk population.

## 1. Introduction

Diabetes, predominantly type 2 diabetes, is one of the leading causes of morbidity and mortality worldwide, affecting over 589 million adults (20–79 years) in 2024 and is predicted to rise to 853 million by 2050 [1]. The Western Pacific region, which includes Australia, is home to over one-third of the global population living with diabetes [1]. Diabetes is a significant public health challenge confronting Australia’s health system, affecting approximately 2 million people (7.5% of the total population) in 2024 and projected to rise to four million in 2050 [2]. Pasifika and other multicultural communities disproportionately bear a higher diabetes burden compared to those of European descent in Australia [3].

Pasifika is a broader term encompassing several sub-ethnic groups with ancestry from the Pacific Islands, including Samoa, Fiji, Tonga, the Cook Islands, and others [4]. Evidence shows that nearly all Pacific Island nations greatly surpass the global average diabetes prevalence [1], while Pasifika populations in high-income countries like the United States and New Zealand face an even greater burden of diabetes compared to the general populations in those countries [5,6].

Australia hosts one of the largest Pasifika populations in the world, due to its proximity to the Pacific Islands. According to the 2021 Australian Census, the Pasifika population was 337,000, accounting for 1.3% of Australia’s total population [7]. Sydney is home to ~25% of Australia’s Pasifika population, with 78.5% of that population concentrated in Western Sydney (the study area) [8]. Despite rapid growth, these populations remain underrepresented in health research. While representative data on diabetes are scarce among Australian Pasifika communities, a small pilot study conducted among Samoans in South Western Sydney found a high prevalence of diabetes (33%) and obesity (77%) [9]. Additionally, an analysis of 2021 Australian Census data indicated elevated rates of self-reported diabetes among individuals of Pasifika ancestry [10]. Moreover, diabetes incidence trends in Australia from 2005 to 2019 and their association with ethnicity found that individuals of Pasifika background had the highest incidence rates of diabetes compared to the general Australian population [3].

The high prevalence of diabetes in Pasifika communities elsewhere is associated with high prevalence of modifiable risk behaviours (physical inactivity, sedentary behaviours, and unhealthy dietary habits) and biomedical risk factors (overweight/obesity, triglycerides) [11]. Additionally, socio-cultural factors that increase diabetes risk, including beliefs such as “big is attractive”, are common in Pasifika communities [12]. These beliefs likely influence dietary habits and participation in weight-reduction activities, ultimately contributing to obesity, diabetes and other chronic health conditions. Limited health literacy and a lack of culturally tailored healthcare services are additional challenges faced by these communities living in Australia [3,12].

In addition to the high prevalence of diabetes, people of Pasifika background in Australia also experience disproportionately high rates of diabetes-related complications. For example, Australian Samoans in Queensland were seven times more likely to be hospitalised due to complications from diabetes compared to the general Australian population in the region, highlighting major gaps in effective diabetes management within these communities [13].

Given the health disparities in Pasifika communities in Australia, understanding the epidemiology of diabetes and its associated factors is vital for developing tailored interventions to address the growing disease burden. However, comprehensive, representative, community-based evidence on the epidemiology of diabetes and its associated risk factors remains limited, particularly evidence derived from active, community-wide health screening across diverse Pasifika communities in Australia. Hence, this cross-sectional analysis was conducted to assess diabetes and associated risk factors among Pasifika adults using baseline data from the Pasifika Preventing Diabetes Programme (PPDP), a community-based stepped-wedge cluster-randomised controlled trial evaluating the effectiveness of a behavioural intervention for diabetes prevention and management [14]. These community-based data from a large sample provide evidence relevant to policymakers, programme implementers, and future researchers interested in preventing and managing diabetes in these high-risk communities.

## 2. Materials and Methods

### 2.1. Study Design

PPDP is a stepped-wedge cluster randomised controlled trial of a behavioural diabetes prevention programme involving 48 churches/communities, each with ≥90% Pasifika congregants/members, located in the Greater Western and South Eastern Sydney regions of New South Wales, Australia (ACTRN12620001101976). Churches/communities are being recruited in “tiers” of 12 churches. Churches are ideal places to reach Pasifika people, as they serve as community hubs where village life is practised, a tradition many have carried with them from their home countries. Recruitment for PPDP occurs continuously, with a target of 70% of congregants/members included in the church/community group registry having baseline data collected from each church/community within the tier before randomisation.

### 2.2. Participants

Pasifika church communities across Greater Western and South Eastern Sydney were mapped through community networks. Adults aged 18 years or older (no maximum age) affiliated with the participating churches, whether through their attendance or that of a family member, were eligible for inclusion in this analysis (Appendix A.1). Churches/communities volunteered to participate through their leadership.

### 2.3. Data Collection

Surveys and physical health screening were conducted at church venues, during home visits, and at community events. Participants were given written or online participant information sheets to review and were encouraged to ask any questions or request clarification before signing a consent form. All clinical data were recorded in a structured form and entered into an online data collection platform, Better Translational Research (BTR; Global Research Systems Australia Pty Ltd, Sydney, Australia). Survey data on clinical, behavioural, and sociodemographic variables were collected via online or paper-based questionnaires. Participants received language support (Tongan, Samoan, and Fijian) from community activators (individuals from Pasifika communities employed by Local Health District Health Promotion and Public Health units or Western Sydney University), who were responsible for recruiting churches and organising data collection and providing cultural support to the participants throughout the research programme as needed.

### 2.4. Sociodemographic and Behavioural Measures

Self-reported socio-demographic variables, including education, age, gender, medical history, alcohol consumption, and smoking history, were collected. Food security was evaluated with a six-item questionnaire adopted from the U.S. Household Food Security Survey Module. The total score was calculated and classified into 0 (food secure), 1 (marginal), 2–4 (moderate food insecurity), and 5–6 (severe food insecurity) [15]. The EQ 5D-3L questionnaire, which assesses participants’ health status across five dimensions (mobility, self-care, usual activities, pain/discomfort, and anxiety/depression, each with three response levels: no problems, some problems and extreme problems) as well as self-rated health on the EQ Visual Analogue Scale (EQ-VAS) from 0 (worst imaginable health) to 100 (best imaginable health), were used to evaluate quality of life. For analysis, responses in each domain were dichotomised into two categories: “no problems” (coded as 0) and “any problems” (coded as 1 and combined the ‘some problems’ and ‘extreme problems’ response categories) [16]. The five-item World Health Organization’s well-being index was employed to evaluate participants’ well-being. The well-being score was derived by summing the individual item scores and was categorised as poor for total scores < 13 and good for scores ≥ 13 [17].

An eight-item fat index validated in Pasifika communities in New Zealand and a 12-item dietary frequency questionnaire assessed nutritional habits. The fat index is a dietary questionnaire-derived measure of fat intake behaviours. It was calculated by dividing the total score for the fat index items by twice the number of items, and expressing the result as a percentage [18]. Fruit and vegetable consumption, which determined whether participants met the minimum daily intake recommendations in Australia (at least 2 servings of fruit and 5 servings of vegetables per day), was assessed using a food frequency questionnaire [19]. The short-form International Physical Activity Questionnaire (IPAQ) assessed physical activity levels, with overall physical activity categorised as low, moderate, or high according to the IPAQ guidelines [20]. Not completing 150 min of moderate-to-vigorous activity over five or more days a week was classified as not meeting the minimum physical activity recommendation [21]. Diabetes knowledge was assessed using a seven-item questionnaire validated in Pasifika communities, with response options of “Yes”, “No”, and “I don’t know”. Incorrect, correct and “I don’t know” were assigned scores of −1, 1 and 0, respectively. The scores were computed, converted to percentages, and categorised into low (<60%), medium (60–79.9%), and high (≥80%) levels based on Bloom’s cut-off [18].

### 2.5. Anthropometric and Clinical Measures

Height was measured to the nearest 0.1 cm using a Seca213 mobile stadiometer (SECA213, Seca GmbH & Co., Hamburg, Germany). Weight was recorded with a digital scale (MS-7301, Charder Electronic Co., Ltd., Taichung City, Taiwan). Body fat percentage was measured using the Tanita BC-402 Smart Family Scale (Tanita BC-402, Tanita Corporation, Tokyo, Japan). Waist circumference was measured with a non-stretch tape meter at the midpoint between the lower margin of the last palpable rib and the top of the iliac crest (right ilium). Weight and waist measurements were recorded twice and averaged for analysis. Body mass index (BMI) was calculated as weight (kg) divided by height squared (m^2^). Polynesian BMI cut-offs were applied to categorise participants into normal (<26 kg/m^2^), overweight (≥26 kg/m^2^ and <32 kg/m^2^), or obese (≥32 kg/m^2^) [22].

Participants’ systolic and diastolic blood pressure were measured in a seated position using a digital blood pressure monitor (Model HBP-1320, OMRON Healthcare, Co., Ltd., Kyoto, Japan) after a rest period of at least 5 min. Two measurements were taken from all participants, two to three minutes apart. A third measurement was taken if the difference between the first and second readings exceeded 20/10 mmHg. The average of the first and second readings, or the first, second, and third readings (where applicable), was used for analysis. Participants were classified as having high blood pressure if they had an average systolic blood pressure of 140 mmHg or higher, an average diastolic blood pressure of 90 mmHg or higher, or a self-reported diagnosis of hypertension [23]. Blood samples were collected from all participants via finger prick to assess HbA1c and random blood glucose using point-of-care testing. HbA1c was measured using the Afinion™ 2 Analyser (Abbott Diagnostics Technologies AS, Oslo, Norway), which has a reportable range of 4.0–15.0% HbA1c with reported total precision coefficients of variation (CVs) generally <3% under controlled conditions [24]. Random blood glucose was measured using Accu-Chek Inform II blood glucose monitors (Roche Diagnostics GmbH, Mannheim, Germany), which have a measuring range of 0.6–33.3 mmol/L and reported repeatability/intermediate precision CVs of approximately 2.0–3.9% across relevant glucose concentrations [25]. Random blood glucose results were reported descriptively only and were not used to define diabetes status. Participants were classified as having previously diagnosed diabetes if they reported a prior diagnosis of diabetes, regardless of their HbA1c level. Those with HbA1c ≥ 6.5% but no previous diabetes diagnosis were considered to have newly identified diabetes. Participants who did not report a prior diabetes diagnosis and had HbA1c < 6.5% were classified as not having diabetes. Prediabetes was defined as HbA1c between 6.0% and 6.4% [26].

The NSW Health Pathology Service provided training, quality assurance and machine maintenance. All clinical measurements were conducted by data collectors received training in data collection methods, including adherence to the developed standard operating procedures SOPs and culturally sensitive approaches. These SOPs were applied consistently across all study sites.

### 2.6. Data Analysis

Data were downloaded from BTR and imported into STATA (version 19.5; StataCorp LLC, College Station, TX, USA) for further data processing and analysis. Missingness was examined for all variables included in the analysis (Appendix A.2). Missing data were minimal for gender, ethnic background, Medicare card ownership, and age, while most sociodemographic and clinical variables had approximately 9–11% missingness. Higher levels of missingness were observed for quality-of-life, physical activity, diet, food security, and diabetes knowledge measures.

Missing data were handled using multiple imputation under the missing-at-random assumption [27]. Forty imputed datasets were generated, and estimates were pooled using Rubin’s rules [28]. The imputation models included all variables reported in the manuscript, including sociodemographic, behavioural, clinical, anthropometric, quality-of-life, diabetes-related variables, and the outcome variable. Two imputation models were fitted: one for analyses involving the full study sample and another for analyses restricted to participants with known diabetes, where diabetes treatment and service utilisation variables were applicable only to this subgroup. Both descriptive and inferential analyses were conducted using the imputed datasets.

Descriptive analyses, including means (arithmetic means for normally distributed data and geometric means for skewed continuous data), proportions, and frequencies, were conducted to summarise the results. Inferential statistics, such as Chi-squared tests for categorical variables and independent t-tests for continuous variables, were used to compare diabetes prevalence across sociodemographic, behavioural, and clinical outcomes. Age-standardised prevalence of diabetes was calculated using the direct standardisation method based on the 2001 Australian standard population [29]. A random-effects meta-analysis was conducted at the church-community level to estimate pooled diabetes prevalence by ethnic background, accounting for potential between-site heterogeneity. Because participants were recruited through churches and community sites, potential clustering at the recruitment-site level was assessed. A random-intercept logistic regression model was fitted to estimate the intraclass correlation coefficient. Multivariable logistic regression analyses were performed to identify statistically significant factors associated with the odds of diabetes. As the intraclass correlation was small, the main regression analyses were repeated using cluster-robust standard errors at the site level to account for possible within-site correlation. The overall fit of the final adjusted model was assessed using the Hosmer–Lemeshow test. Multicollinearity among the predictor variables was assessed using variance inflation factors (VIFs). Complete-case analyses were also performed as sensitivity analyses to assess the robustness of the findings. Statistical significance was determined using a *p*-value < 0.05 and corresponding 95% confidence intervals (95% CI).

## 3. Results

### 3.1. Sociodemographic and Behavioural Characteristics of the Participants

The present study is a cross-sectional analysis of baseline data collected from 1161 Pasifika adults across 45 out of 48 (93.8%) churches/communities. Table 1 presents sociodemographic and behavioural characteristics of the study participants by diabetes status. The mean age of the participants was 46.7 ± 15.6 years. Females constituted 53.2% of the sample. The majority identified as Tongan (68.2%), followed by Fijian (19.2%). Additionally, 78.3% had attained secondary education or higher. Participants with diabetes were more likely to be older, female, identify as Tongan, have lower levels of education, be unemployed, have longer residency in Australia, report a family history of diabetes, report existing comorbidities, have a general practitioner, and experience difficulties with mobility and usual activities.

Over two-third (68.9%), of participants reported insufficient levels of physical activity, nearly all (97.4%) did not meet the minimum recommendations for fruit and vegetable intake, and half (50.5%) had limited knowledge of diabetes. More than two-thirds (69.5%) of participants reported some level of food insecurity, with 58.3% experiencing moderate-to-severe and 11% marginal. While there was no significant difference in fruit and vegetable intake or food insecurity status, participants with diabetes were less likely to have sufficient physical activity level, engage in vigorous physical activity, and high-fat dietary habits (Table 1).

### 3.2. Clinical Characteristics of Participants

**Figure 1:**
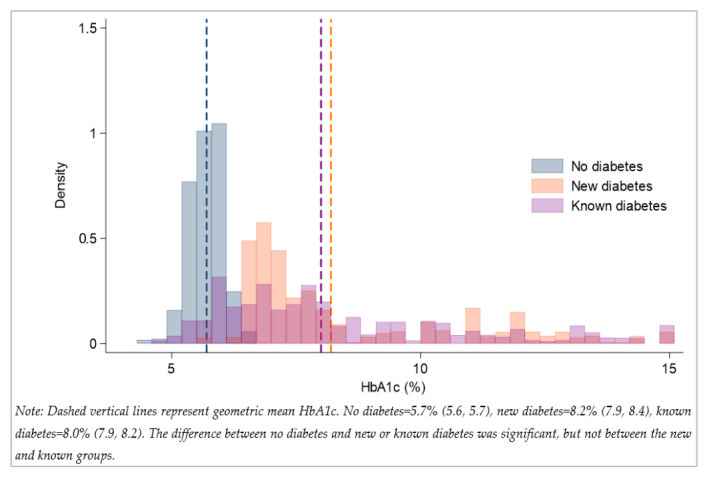
HbA1c distribution by diabetes status among Pasifika adults (broken lines show the corresponding geometric means). Estimates were based on multiply imputed data; missingness in the original observed data is presented in Appendix A.2.

Overall, 95.8% of the participants were overweight/had obesity and 68.0% had high blood pressure. Geometric mean HbA1c was higher among participants with newly identified diabetes (65.7 mmol/mol [8.2%]; 95% CI: 63.0–68.3 mmol/mol) and those with known diabetes (64.4 mmol/mol [8.0%]; 95% CI: 62.3–66.5 mmol/mol) compared with individuals without diabetes (38.3 mmol/mol [5.7%]; 95% CI: 37.5–38.9 mmol/mol) (*p* < 0.001), with no significant difference between the two diabetes groups (Figure 1). Participants with diabetes had higher waist circumference, body fat percentage, pulse rate, systolic blood pressure, random blood glucose and HbA1c than those without diabetes (Table 2).

### 3.3. The Prevalence of Diabetes

**Figure 2:**
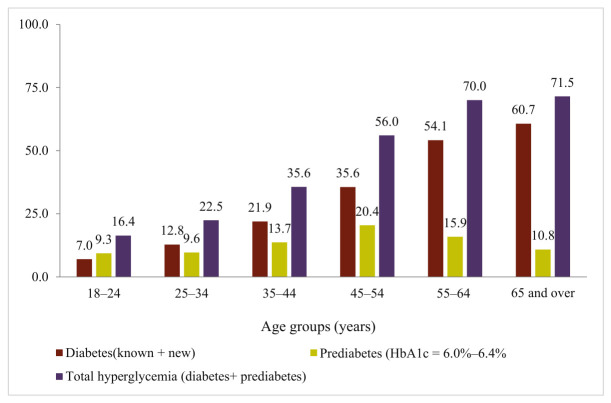
Prevalence of prediabetes and diabetes across age groups among Pasifika adults in Sydney (n = 1161). Estimates were based on multiply imputed data; missingness in the original observed data is presented in Appendix A.2.

**Figure 3:**
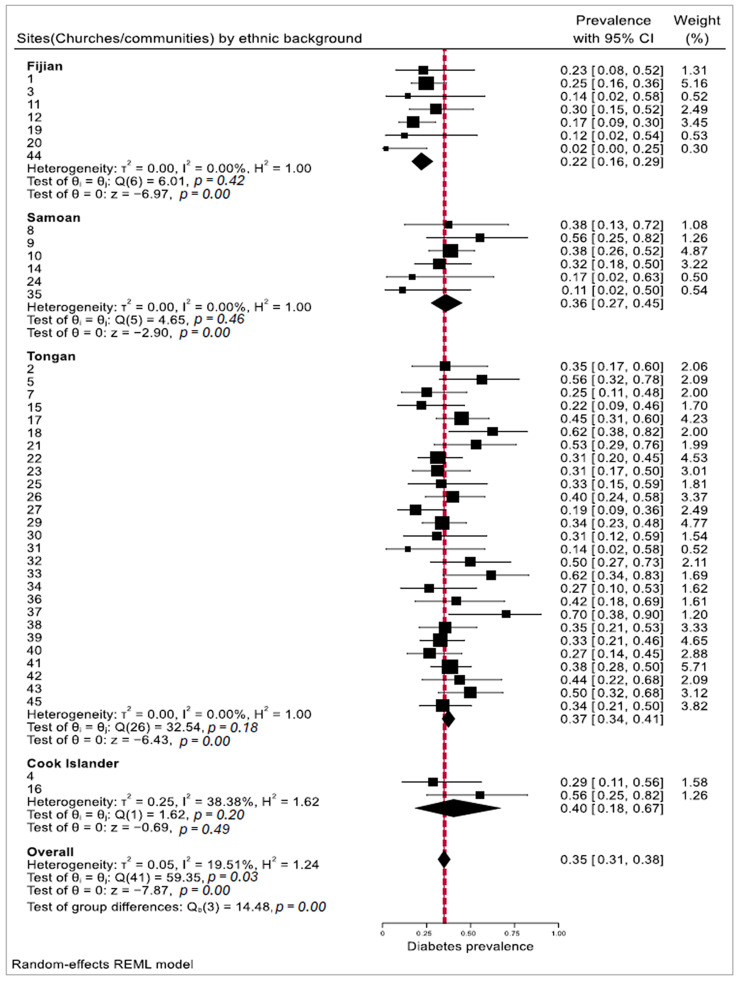
Subgroup meta-analysis of diabetes prevalence across church/community sites by Pasifika ethnic background (Each square represents the estimated prevalence of diabetes for an individual church/community site, and the horizontal line represents the 95% confidence interval. The size of the square reflects the relative weight of each site in the analysis. Diamonds show the pooled prevalence estimates for each ethnic subgroup and the overall sample. Pooled estimates were calculated using a random-effects restricted maximum-likelihood model. Heterogeneity statistics are presented for each subgroup and overall. The test of group differences compares pooled diabetes prevalence across ethnic subgroups).

The overall crude prevalence of diabetes, encompassing both newly identified cases (11.6%) and previously known cases (22.3%), was 33.9% (95% CI: 31.1–36.7%). The crude prevalence of prediabetes was 14.2% (95% CI: 12.0–16.3%). After age standardisation, the prevalence of diabetes was 31.2% (95% CI: 28.8–33.7%), and that of prediabetes was 13.4% (95% CI: 11.4–15.3%). The prevalence of diabetes increased significantly with age, rising from 7.0% in the 18–24-year age group to 60.8% in those aged ≥65 years (Figure 2), and varied across church/community sites, ranging from 0% to 62.5% (Figure 3). The proportion of diabetes cases that were newly identified varied by age group, with the highest proportions among participants aged 18–24 years (70.6%) and 35–44 years (60.7%), and the lowest among those aged 55–64 years (22.1%) and 65 years and over (23.8%). More than one-fourth, 27.1% (314), of the participants had both high blood pressure and diabetes, and 74.7% (869) had either diabetes or high blood pressure.

In a random-effects meta-analysis, the pooled prevalence of diabetes was 35% (95% CI: 31–38). The pooled prevalence was lowest among Fijian church communities at 22% (95% CI: 16–29), followed by Samoan at 36% (95% CI: 27–45), Tongan at 37% (95% CI: 34–41), and Cook Islander at 40% (95% CI: 18–67). There was evidence of statistically significant subgroup differences by ethnic background, Q(3) = 14.48, *p* < 0.001. Within-subgroup heterogeneity was low for the Fijian, Samoan, and Tongan groups, whereas the Cook Islander subgroup showed moderate heterogeneity, but was based on only 2 sites. Overall heterogeneity was low to modest (I^2^ = 19.5%; Figure 3).

### 3.4. Diabetes Management, Treatment Guideline Achievement

Among those who had known diabetes, the mean age at first diagnosis was 43.6 ± 21.4 years, with an average duration of diabetes of 10.6 years. The majority of participants, 65.9% (n = 224), reported taking tablets; 15.8% (n = 54) reported taking no medication; 11.5% (n = 39) reported using both insulin and tablets; 6.8% (n = 23) reported using insulin; and 4.7% (n = 16) reported taking GLP-1 receptor agonists. About one-third (32.8%; n = 112) of the participants reported not checking their blood sugar at least once a month. Regarding blood glucose monitoring methods, 33.3% (n = 113) of participants reported monitoring their blood sugar at home, 21.1% (n = 73) checked it at their doctor’s or educator’s office, 25.4% (n = 86) used a sensor, and 20.3% (n = 69) were not monitoring their blood sugar at all.

Regarding diabetes healthcare utilisation, 69.3% (n = 236) reported having visited their general practitioner at least five times in the past year, whereas 82.2% (n = 280) reported not attending a hospital diabetes clinic. In the year before the survey, 41.1% (n = 140) and 31.9% (n = 108) reported having eye and foot check-ups, respectively. Only 44.9% (153), 26.4% (n = 90), and 13.9% (n = 47) met treatment targets for glycaemia (HbA1c ≤ 7.0), blood pressure (<140/90 mmHg), and for both, respectively.

### 3.5. Factors Associated with Diabetes

Table 3 presents the results of a bivariable and multivariable logistic regression analysis of factors associated with the odds of having diabetes. In the bivariable analyses, several sociodemographic, behavioural, and clinical factors were associated with diabetes. In the multivariable model, older age remained independently associated with increased odds of diabetes, with each additional year of age linked to a 6% increase in the odds of diabetes. Participants with a family history of diabetes had more than twice the odds of diabetes compared with those without a family history, and those with high blood pressure had higher odds of diabetes than those with normal blood pressure. In contrast, participants reporting a high level of physical activity had 41% lower odds of diabetes than those with low physical activity. Other variables were not significantly associated with diabetes after adjustment.

The intraclass correlation coefficient (ICC) estimated from the random-intercept logistic regression model was 0.025, indicating limited site-level variation in diabetes status. Given the small ICC, the primary multivariable logistic regression model was repeated using cluster-robust standard errors at the site level to account for potential within-site correlation. The findings were materially unchanged after accounting for clustering. In addition, a complete-case sensitivity analysis was conducted to assess the robustness of the multiple-imputation findings. Older age was associated with higher odds of diabetes in both models, while a high physical activity level was associated with lower odds of diabetes. Most sociodemographic, behavioural, and anthropometric variables were not independently associated with diabetes in either model. However, some differences in statistical significance were observed. Family history of diabetes and high blood pressure were statistically significant in the multiple-imputation model but not in the complete-case model (Table A2). Overall, the findings were broadly consistent in direction, but the multiple-imputation model retained the full analytical sample and may provide more precise estimates under the missing-at-random assumption.

## 4. Discussion

The study found that the prevalence of diabetes (33.9%) was high among Pasifika adults living in Sydney (31.2% age-standardised), a prevalence five times higher than in the general Australian adult population (6.6%) [30]. A further 14.2% (13.4% age-standardised) of adults had prediabetes, suggesting that approximately one in two Pasifika adults were affected by hyperglycaemia (and potentially more given the limited sensitivity of HbA1c as a diabetes screening test) [31]. Known diabetes risk factors, including low fruit and vegetable intake, physical inactivity and obesity, were prevalent, as was high blood pressure. Although the odds of having diabetes did not differ significantly across several socio-demographic, behavioural, and clinical characteristics of the participants, it was positively and significantly associated with older age, a family history of diabetes, and high blood pressure. Conversely, the odds of having diabetes were significantly lower among participants with high levels of physical activity.

This is the first analysis to involve a large sample and report on more than a single Pasifika group, assessing diabetes and associated risk factors in Pasifika communities living in Australia. The observed prevalence of diabetes (33.9%) is comparable to the prevalence (33%) reported by a pilot study conducted among Samoans living in Western Sydney attending different churches [32]. However, our finding is higher than findings reported in similar populations in the USA (18.3%) and in New Zealand (5.3–15.4%) [33,34]. Similarly, the prevalence of diabetes among our sample is higher than in Pacific Island nations (12.0–25.7%) [1]. Consistent with earlier studies, the age-standardised prevalence of diabetes among our sample of Pasifika adults is about five times higher than that of the general Australian adult population (31.2% vs. 6.6%) [30,35]. The age-specific distribution of diabetes shows a markedly elevated prevalence among our younger Pasifika sample, with prevalence in those aged 45–54 years (35.6%) exceeding that observed in the general Australian population aged 75 years and older (15%) by more than twofold [30]. Furthermore, the age-standardised prevalence of undiagnosed diabetes is ten times higher among our sample compared to the general Australian population (11.2% vs. 1.0%) [30]. The average HbA1c among participants newly identified with diabetes was well above the diagnostic threshold, suggesting that many cases may reflect delayed diagnosis rather than borderline hyperglycaemia. These findings highlight potential gaps in routine diabetes screening and support the need for culturally safe, community-based pathways to promote earlier detection and linkage to ongoing care.

Although diabetes is generally more common with increasing age, younger participants with diabetes were more likely to be previously undiagnosed. To our knowledge, this pattern has not previously been reported among Pasifika communities in Australia. However, similar evidence from a New Zealand population-based study, which included both Pacific and Māori participants, showed that among adults aged <45 years, a substantial proportion of diabetes was undiagnosed. These findings suggest that younger high-risk Pasifika adults may be missed by current screening approaches. The high prevalence of type 2 diabetes in this Pasifika sample may be attributed to a multifactorial interplay of genetic and social determinants. Previous studies have reported genetic susceptibility to diabetes among Polynesian populations; however, evidence such as for CREBRF rs373863828 allele, which is associated with higher BMI but lower type 2 diabetes risk, suggests that genetic factors alone are unlikely to explain the observed diabetes burden [36]. As documented elsewhere, broader structural and sociocultural factors, including limited health literacy, barriers to culturally appropriate healthcare, post-migration dietary changes, and reduced physical activity, may also contribute [12,36,37,38]. This finding underscores the need for culturally safe, multilevel strategies for diabetes prevention and management within Pasifika communities.

In bivariable analysis, diabetes prevalence differed across Pasifika subgroups, with Tongan participants showing a higher prevalence compared to Fijian participants, consistent with patterns reported in the 2021 Australian Census, which showed a 5% higher diabetes prevalence among Tongans [10]. Although subgroup differences were observed in the bivariable analysis, they should be interpreted with caution because some ethnic groups were underrepresented, and ethnic background was not independently associated with diabetes in the fully adjusted model. This longstanding disparity may be partly attributed to differences in the distribution of genetic susceptibility variants, which have been reported to be more common in Polynesian than in Melanesian populations [39]. However, current evidence is too limited to conclude whether genetic factors alone can explain Pasifika subgroup differences in diabetes prevalence. From this perspective, sociocultural factors may play a significant role in explaining the disparity. For instance, greater English proficiency among Fijians, linked to historical ties to the Commonwealth, may facilitate access to health information, improve health literacy, and enhance engagement with health services in English-speaking settings such as Australia [12]. These results support the need for culturally and linguistically tailored diabetes prevention efforts that acknowledge diversity within Pasifika communities and address upstream determinants of health. Larger studies with more balanced representation across Pasifika subgroups are needed to better understand these differences.

In line with the existing literature, the known risk factors associated with diabetes were associated with increased odds of diabetes in our analysis, where participants with a family history of diabetes [40], older age [41] and high blood pressure [42] were more likely to have diabetes, highlighting the importance of considering these disparities when developing diabetes prevention strategies for Pasifika communities.

BMI, body fat percentage, and waist circumference were not independently associated with diabetes in the adjusted model. This finding should be interpreted in the context of the very high prevalence of overweight/obesity and central obesity in the study population, which may have limited the ability of adiposity measures to distinguish between participants with and without diabetes. Therefore, the absence of an independent association should not be interpreted as evidence that adiposity is not relevant to diabetes risk. Rather, it may reflect a high background burden of obesity-related risk in this population. In contrast, a high level of physical activity was associated with 41% lower odds of diabetes. While this association has not previously been reported among Pasifika populations residing in Western countries, comparable findings have been reported in prior studies involving Polynesian and Indian men in Fiji and adults in Western Samoa [43,44]. Notably, although a substantial proportion of participants did not meet the recommended minimum threshold for physical activity, most indicated either an intention to increase activity or making active efforts to do so. These findings suggest that culturally tailored health promotion strategies, including community-based physical activity programs, warrant further investigation as components of diabetes prevention and management. Given the prevailing obesogenic environment and structural barriers such as food insecurity, integrating physical activity interventions with dietary strategies, along with improving access to nutritious foods, may be important considerations for future diabetes prevention and management efforts [45].

More than two-thirds (69.5%) of the study participants reported experiencing food insecurity, a prevalence markedly higher than the 32% observed in the general Australian population [46] and substantially greater than rates reported among Pacific Islanders in the United States [47] and New Zealand [48]. The observed discrepancy may stem from variations in measurement approaches, as prior studies used different instruments to assess food security. This finding carries substantial public health significance, given that food insecurity is strongly associated with poor dietary quality, obesity, and an increased risk of chronic conditions such as diabetes [49]. Moreover, in light of the high prevalence of diabetes, food insecurity may exacerbate hyperglycaemia and diabetes-related complications by limiting access to healthy diets, which are integral to effective diabetes management. Consequently, implementing culturally tailored, community-based strategies to enhance food access among populations at elevated risk would represent a critical and beneficial intervention.

Among participants with known diabetes, the findings highlight important gaps in ongoing diabetes management. Although many participants reported frequent contact with general medical practitioners, this did not consistently translate into optimal monitoring, screening for complications, or the achievement of treatment targets. Less than half achieved the glycaemic target, approximately one-quarter achieved the blood pressure target, and only 13.9% achieved both targets. While glycaemic target achievement was higher than the national average, blood pressure control was lower than national estimates [50]. Both indicators were also very low compared to the World Health Organization Global Diabetes Compact targets for 2030, which aim for 80% of people with diabetes to achieve glycaemic control and 80% to achieve blood pressure control [51]. These findings suggest that improving diabetes outcomes in Pasifika communities may require stronger systems for ongoing risk-factor control, treatment optimisation, and regular monitoring. The particularly low proportion achieving both glycaemic and blood pressure targets highlights the need for culturally safe, proactive, and coordinated models of care to support long-term diabetes management and reduce preventable cardiovascular and microvascular complications.

Although most participants reported using glucose-lowering medication, a notable proportion reported no such use, limited glucose monitoring, and very low use of GLP-1 receptor agonists, despite the high burden of obesity in this population. These findings should be interpreted cautiously, as treatment choices and glucose monitoring need to be individualised. However, they point to the need for more accessible, culturally safe, and coordinated models of diabetes care that strengthen treatment optimisation, self-management support, complication screening, and linkage between primary care, diabetes educators, and specialist services.

### Strengths and Limitations of the Study

Strengths of this study include being the first to estimate diabetes prevalence among broader Pasifika communities in Australia, using a relatively large sample and community-based primary data collection methods. This approach enabled the identification of previously undiagnosed cases, providing a more accurate picture of disease burden. The multiple-imputation model retained the full analytical sample and may provide more precise estimates under the missing-at-random assumption. This study also has several limitations. Diabetes status was based on a single HbA1c measurement and self-reported previous diagnosis, without confirmation using Oral Glucose Tolerance Test (OGTT), fasting plasma glucose, repeat HbA1c testing, venous laboratory testing, or medical record review. As a result, misclassification of diabetes status may have occurred, particularly among participants identified as having newly identified diabetes based on HbA1c ≥ 6.5% alone. Although OGTT may be more sensitive for detecting diabetes, it was not feasible in this community-based screening study because of practical challenges, including fasting requirements and fieldwork logistics [31]. In addition, this study may not provide a comprehensive cardiometabolic risk profile, as lipid parameters (including cholesterol and triglycerides), renal markers (eGFR and albuminuria), inflammatory markers, and validated cardiovascular/metabolic risk scores were not available. The cross-sectional nature of the study limits causal inference and allows for the possibility of reverse causation, particularly in the association between diabetes and physical activity, as people with diabetes may be less physically active due to their health status. Although multiple imputation was used to minimise potential bias from missing data, some behavioural and quality-of-life variables still had substantial levels of missingness. Therefore, findings related to physical activity, diet, food security, diabetes knowledge, and quality of life should be interpreted with appropriate caution, as missing data may have affected the precision and reliability of these estimates. While the church-based sampling approach was valuable for engaging a high-risk and underrepresented population, it may have limited the generalisability of the findings. Pasifika adults connected with churches or community networks may differ from those who are less connected to these settings. The sample also included a higher proportion of Tongan participants, with smaller representation from some other Pasifika groups, including Cook Islanders. Therefore, the findings are most applicable to Pasifika adults reached through church and community networks, and should not be interpreted as fully representative of all Pasifika adults in Australia. Finally, several behavioural and psychosocial measures, including physical activity, diet, alcohol intake, smoking/vaping, quality of life and well-being, diabetes knowledge, and food insecurity, were self-reported, which may be affected by recall bias, social desirability bias, and misclassification.

## 5. Conclusions

This study identified a high prevalence of diabetes among Pasifika adults, with a substantial proportion of cases previously undiagnosed and newly identified through community-based screening. This suggests important gaps in the timely detection of diabetes and highlights the value of culturally safe screening pathways within Pasifika communities. This was accompanied by very low engagement in preventive health behaviours, a markedly high prevalence of high blood pressure, and widespread overweight and obesity. A high level of physical activity was associated with lower odds of diabetes. These findings underscore the urgent need for culturally tailored strategies to enhance awareness, improve diabetes screening uptake, and strengthen diabetes prevention and management within Pasifika communities in Australia. The findings also provide evidence to inform future interventions targeting physical activity and broader structural factors that are relevant to the burden of type 2 diabetes and its complications in this high-risk population.

## Figures and Tables

**Table 1:** Socio-demographic and behavioural characteristics of Pasifika adults by diabetes status.

Variables		Overall (n = 1161)	Diabetes		*p*-Value
			No (n = 768)	Yes (n = 393)	
Age (years), (mean ± SD)		46.7 ± 15.6	42.3 ± 14.9	55.2 ± 13.2	**<0.001**
	Female, n (%)	618 (53.2)	395 (51.5)	223 (56.7)	0.101
Ethnic background, n (%)					
	Fijian	223 (19.2)	174 (22.7)	49 (12.3)	**<0.001**
	Samoan	120 (10.3)	77 (10.0)	43 (11.0)	
	Tongan	792 (68.2)	501 (65.2)	291 (74.2)	
	Cook Islander	26 (2.2)	16 (2.1)	10 (2.5)	
Education (secondary or higher), n (%)		909 (78.3)	617 (80.4)	291 (74.1)	**0.025**
Currently employed, n (%)		840 (72.3)	607 (79.1)	233 (59.2)	**<0.001**
Duration in Australia (years), (mean ± SD)		19.1 ± 14.3	16.9 ± 13.1	23.5 ± 15.4	**<0.001**
Had a family history of diabetes, n (%)		470 (40.5)	264 (34.4)	206 (52.2)	**<0.001**
Had Medicare card, n (%) ^α^		617 (53.1)	394 (51.3)	223 (56.7)	0.37
Had a GP, n (%)		492 (42.3)	287 (37.4)	205 (52.0)	**<0.001**
Self-reported co-morbidities, n (%)		364 (31.3)	174 (22.7)	190 (48.2)	**<0.001**
Self-rated health state score (mean ± SD)		60.5 ± 24.3	60.9 ± 24.7	59.7 ± 23.5	0.597
Had mobility problems, n (%)		165 (14.2)	73 (9.5)	92 (23.3)	**<0.001**
Had personal care problems, n (%)		73 (6.3)	42 (5.5)	32 (8.0)	0.230
Had problems performing daily activities (%)		103 (8.9)	45 (5.8)	59 (14.9)	**<0.001**
Experienced pain/discomfort, n (%)		300 (25.8)	196 (25.6)	103 (26.3)	0.863
Felt anxious/depressed, n (%)		159 (13.7)	101 (13.2)	58 (14.7)	0.577
Good wellbeing, n (%)		998 (86.0)	655 (85.4)	343 (87.1)	0.535
Engaged in moderate physical activities		561 (48.3)	381 (49.6)	180 (45.7)	0.325
Engaged in vigorous physical activities		531 (45.8)	379 (49.4)	152 (38.7)	**0.008**
Met the Australian physical activity recommendation, n (%)		398 (34.3)	297 (38.7)	101 (25.7)	**<0.001**
Met fruit & vegetable recommendations, n (%)		29 (2.5)	21 (2.7)	8 (2.1)	0.600
Fat index percentage, mean ± SD		38.3 ± 10.4	37.1 ± 10.6	40.7 ± 9.7	**<0.001**
Food security category, n (%)					
Food secure		355 (30.6)	229 (29.8)	126 (32.1)	0.768
Marginal food insecurity		129 (11.1)	86 (11.2)	43 (10.9)	
Moderate food insecurity		392 (33.8)	256 (33.3)	136 (34.6)	
Severe food insecurity		285 (24.5)	197 (25.7)	88 (22.4)	
Drink alcohol, n (%)		351 (30.2)	250 (32.6)	100 (25.5)	**0.019**
Smoke/vape, n (%)		307 (26.5)	227 (29.6)	80 (20.4)	**0.002**
Diabetes knowledge (medium/high), n (%)		557 (48.0)	354 (46.1)	203 (51.7)	0.236

^α^ Medicare is Australia’s universal health insurance scheme to help Australians with the costs of their healthcare, regardless of where they live or their ability to pay. GP: General medical practitioner; SD: Standard deviation; Bolded denotes a significant difference, *p* < 0.05. Estimates were based on multiply imputed data; missingness in the original observed data is presented in Appendix A.2.

**Table 2:** Clinical outcomes among Pasifika adults living in Sydney by diabetes status.

Variables	Overall (n = 1161)	Without Diabetes (n = 768)	With Diabetes (n = 393)	*p*-Value
Average weight (kg), mean ± SD	109.4 ± 24.5	109.4 ± 23.4	109.5 ± 26.6	0.937
Height (cm), mean ± SD	1.7 ± 0.1	1.7 ± 0.1	1.7 ± 0.1	**0.003**
Waist circumference (cm), mean ± SD	117.2 ± 21.6	116.0 ± 24.2	119.5 ± 15.2	**0.020**
Had central obesity, n (%)	1079 (93.0)	698 (90.9)	381 (96.9)	**0.001**
Body fat percentage, mean ± SD	38.3 ± 10.4	37.1 ± 10.6	40.7 ± 9.7	**<0.001**
BMI (kg/m^2^), GM (95% CI)	36.7 (36.3–37.2)	36.5 (35.9–37.1)	37.1 (36.3–37.9)	0.223
Normal, n (%)	49 (4.2)	36 (4.7)	12 (3.1)	
Overweight, n (%)	239 (20.6)	151 (19.7)	88 (22.4)	0.312
Obese, n (%)	873 (75.2)	580 (75.6)	293 (74.5)	
Heart rate (BPM), mean ± SD	78.6 ± 14.3	77.9 ± 14.7	79.8 ± 14.1	**0.038**
SBP (mmHg), mean ± SD	143.8 ± 22.2	141.7 ± 20.6	148.0 ± 24.6	**<0.001**
DBP (mmHg), mean ± SD	87.8 ± 12.8	88.2 ± 12.3	87.2 ± 13.9	0.234
High blood pressure (%)	790 (68.0)	476 (62.0)	314 (79.8)	**<0.001**
RBG (mmol/L), GM (95% CI)	6.8 (6.7–7.0)	5.9 (5.7–6.0)	9.2 (8.9–9.5)	**<0.001**
HbA1c (%), GM (95% CI)	6.4 (6.3–6.5)	5.7 (5.6–5.7)	8.1 (7.9–8.2)	**<0.001**

BMI: Body mass index, BPM: Beat Per Minute, CI: Confidence interval, DBP: Diastolic Blood Pressure, GM: Geometric mean, HbA1c: Glycated haemoglobin, RBG: Random Blood Glucose, SBP: Systolic Blood Pressure, SD: Standard Deviation, Bolded denotes a significant difference, *p* < 0.05. Estimates were based on multiply imputed data; missingness in the original observed data is presented in Appendix A.2.

**Table 3:** Factors associated with diabetes among Pasifika adults in Sydney.

Variables		COR (95% CI)	*p*-Value	AOR (95% CI)	*p*-Value
Age (per year)		1.06 (1.05, 1.07)	**<0.001**	1.06 (1.05, 1.07)	**<0.001**
Gender					
	Female (ref)	1.00		1.00	
	Male	0.81 (0.63, 1.04)	0.101	1.12 (0.75, 1.66)	0.588
Ethnic background					
	Fijian (ref)	1.00		1.00	
	Samoan	2.02 (1.21, 3.39)	**0.008**	1.27 (0.68, 2.38)	0.455
	Tongan	2.10 (1.44, 3.06)	**<0.001**	1.51 (0.94, 2.42)	0.086
	Cook Islander	2.25 (0.95, 5.32)	0.065	0.66 (0.24, 1.79)	0.411
Educational attainment (per level)		0.84 (0.77, 0.93)	**0.001**	0.89 (0.80, 1.00)	0.058
Duration in Australia (per year)		1.03 (1.02, 1.04)	**<0.001**	1.00 (0.99, 1.01)	0.830
Had Medicare ^α^					
	No (ref)	1.00		1.00	
	Yes	1.25 (0.96, 1.61)	0.095	1.16 (0.82, 1.63)	0.402
Had a GP					
	No (ref)	1.00		1.00	
	Yes	1.81 (1.40, 2.35)	**<0.001**	1.01 (0.72, 1.43)	0.937
Family history of Diabetes					
	No (ref)	1.00		1.00	
	Don’t know/unsure	0.93 (0.62, 1.38)	0.705	1.07 (0.66, 1.72)	0.794
	Yes	2.04 (1.52, 2.72)	**<0.001**	2.23 (1.56, 3.20)	**<0.001**
Physical activity level					
	Low (ref)	1.00		1.00	
	Moderate	0.81 (0.56, 1.18)	0.279	0.73 (0.47, 1.13)	0.154
	High	0.50 (0.36, 0.72)	**<0.001**	0.59 (0.38, 0.94)	**0.026**
Fruit intake (serves per week)		0.99 (0.97, 1.02)	0.702	1.00 (0.97, 1.03)	0.952
Vegetable intake (serves per week)		0.99 (0.97, 1.01)	0.213	1.00 (0.98, 1.02)	0.873
Fat index		0.99 (0.99, 1.00)	**0.026**	1.00 (0.99, 1.01)	0.502
Food security					
	Food secure	1.00		1.00	
	Marginal food insecurity	0.88 (0.52, 1.50)	0.642	0.98 (0.53, 1.82)	0.952
	Moderate food insecurity	0.97 (0.67, 1.41)	0.863	0.96 (0.61, 1.53)	0.876
	Severe food insecurity	0.81 (0.53, 1.24)	0.325	0.88 (0.52, 1.50)	0.636
Drink alcohol					
	No (ref)	1.00		1.00	
	Yes	0.71 (0.53, 0.94)	**0.019**	1.01 (0.70, 1.45)	0.961
Smoke/vape					
	No (ref)	1.00		1.00	
	Yes	0.61 (0.45, 0.83)	**0.002**	0.97 (0.65, 1.44)	0.882
Diabetes knowledge (per %)		1.01 (1.00, 1.01)	0.164	1.00 (0.99, 1.01)	0.595
Waist circumference (per 1 cm)		1.01 (1.00, 1.02)	**0.020**	1.00 (1.00, 1.01)	0.520
Body fat percentage (per %)		1.03 (1.02, 1.05)	**<0.001**	1.02 (0.99, 1.04)	0.161
Body mass index (per 1 kg)		1.01 (0.99, 1.02)	0.247	1.01 (0.98, 1.03)	0.655
Blood Pressure					
	Normal (ref)	1.00		1.00	
	High blood pressure	2.42 (1.77, 3.32)	**<0.001**	1.65 (1.15, 2.38)	**0.007**

Note, AOR: Adjusted odds ratio, COR: Crude odds ratio, GP: General practitioner, ref: reference, Bolded denotes a significant difference, *p* < 0.05. Variance inflation factor (VIF) < 5 for all variables. ^α^ Medicare is Australia’s universal health insurance scheme to help Australians with the costs of their healthcare, regardless of where they live or their ability to pay. Estimates were based on multiply imputed data; missingness in the original observed data is presented in Appendix A.2.

**Figure A1:**
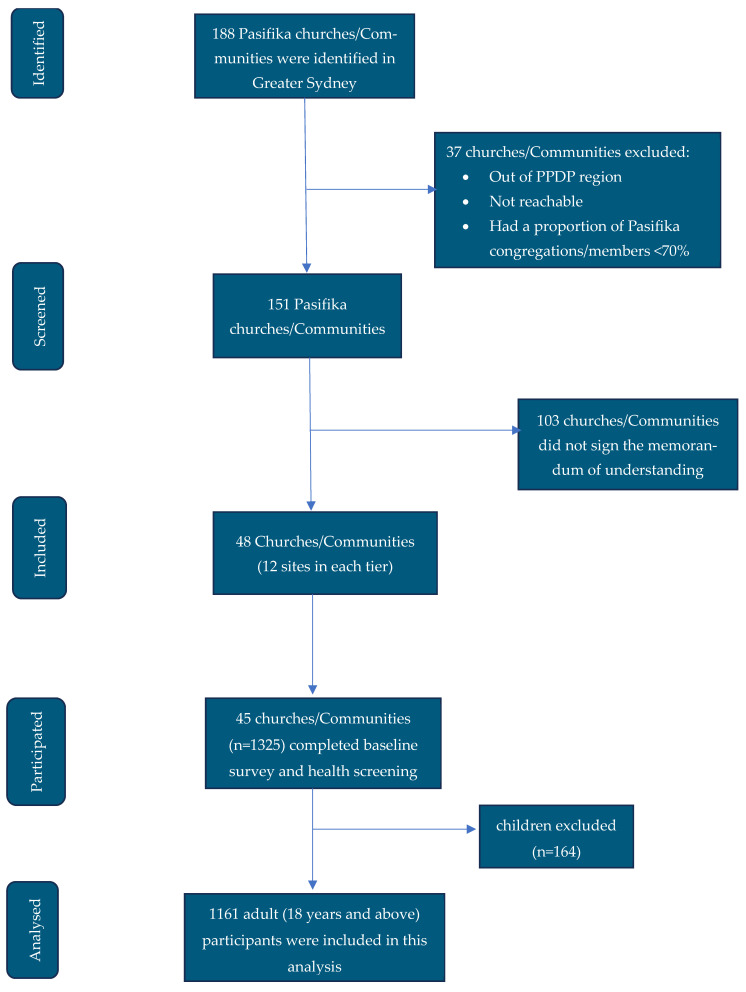
Sampling diagram (PPDP: Pasifika Preventing Diabetes Programme).

## Data Availability

The data presented in this study are available on request from the corresponding author because the data are part of an ongoing trial.

## Abbreviations

The following abbreviations are used in this manuscript:

AOR	Adjusted Odds Ratio
BMI	Body Mass Index
BTR	Better Translational Research
CI	Confidence Interval
COR	Crude Odds Ratio
DBP	Diastolic Blood Pressure
EQ 5D-3L	EuroQol Five-Dimension Three-Level questionnaire
EQ-VAS	EuroQol Visual Analogue Scale
GM	Geometric Mean
GP	General Practitioner
HbA1c	Glycated Hemoglobin
IPAQ	International Physical Activity Questionnaire
PPDP	Pasifika Preventing Diabetes Programme
RBG	Random Blood Glucose
REML	Restricted Maximum Likelihood
SBP	Systolic Blood Pressure
SD	Standard Deviation
VIF	Variance Inflation Factor

## Appendix A

### Appendix A.2

**Table A1:** Missingness summary of key variables included in the analysis (n = 1161).

Variables	Observed	Missing	Missingness (%)
Gender		0	0.0
Age	1101	60	5.2
Education	1042	119	10.2
Ethnic background		0	0.0
Working status	1035	126	10.9
Duration in Australia	1042	119	10.2
Alcohol	1042	119	10.2
Smoking/vaping	1042	119	10.2
Family history of diabetes	1042	119	10.2
Medicare card ownership	1134	27	2.3
Having your own GP	1042	119	10.2
Comorbidity	1050	111	9.6
EQ-VAS	567	594	51.2
EQ-5D-3L	567	594	51.2
Quality of life	567	594	51.2
Physical activity	610	551	47.5
Diet	620	541	46.6
Food security	511	650	56.0
Diabetes knowledge	619	542	46.7
Ever told to have diabetes	1053	108	9.3
Ever told to have high blood pressure	1042	119	10.2
Weight	1042	119	10.2
Height	1043	118	10.2
Waist	1036	125	10.8
Body fat	933	228	19.6
Heart rate	1031	130	11.2
Systolic blood pressure	1049	112	9.6
Diastolic blood pressure	1049	112	9.6
Random blood glucose	1032	129	11.1
HbA1c	1031	130	11.2
Overall	310	851 *	73.3

* The number of participants with missing data for at least one of the variables.

### Appendix A.3

**Table A2:** Logistic regression analysis of factors associated with diabetes status (Complete case analysis).

Variables	COR (95% CI)	*p*-Value	AOR (95% CI)	*p*-Value
Age (per year)	1.067 (1.056–1.079)	<0.001	1.087 (1.056–1.118)	<0.001
Gender				
Female	Ref	-	Ref	-
Male	0.780 (0.606–1.003)	0.052	0.670 (0.287–1.567)	0.356
Ethnic background				
Fijian	Ref	-	Ref	-
Samoan	2.049 (1.232–3.408)	0.006	0.692 (0.092–5.232)	0.722
Tongan	2.210 (1.529–3.195)	<0.001	1.701 (0.573–5.049)	0.339
Cook Islander	2.530 (1.071–5.974)	0.034	1.149 (0.093–14.143)	0.914
Educational attainment (per level)	0.838 (0.763–0.921)	<0.001	0.849 (0.686–1.050)	0.131
Duration in Australia (per year)	1.035 (1.025–1.045)	<0.001	0.984 (0.959–1.009)	0.201
Had Medicare				
No	Ref	-	Ref	-
Yes	1.257 (0.975–1.619)	0.077	1.149 (0.593–2.225)	0.681
Had a GP				
No	Ref	-	Ref	-
Yes	1.896 (1.457–2.467)	<0.001	0.980 (0.483–1.988)	0.956
Family history of diabetes				
No	Ref	-	Ref	-
Don’t know/unsure	0.899 (0.605–1.336)	0.598	0.649 (0.238–1.768)	0.398
Yes	2.087 (1.561–2.790)	<0.001	1.829 (0.939–3.561)	0.076
Physical activity level				
Low	Ref	-	Ref	-
Moderate	0.795 (0.509–1.243)	0.315	0.597 (0.275–1.297)	0.193
High	0.426 (0.284–0.639)	<0.001	0.447 (0.218–0.916)	0.028
Fruit intake (serves per week)	0.993 (0.966–1.021)	0.619	0.939 (0.846–1.043)	0.241
Vegetable intake (serves per week)	0.981 (0.961–1.001)	0.068	1.001 (0.969–1.034)	0.952
Fat index	0.989 (0.983–0.996)	0.001	0.990 (0.979–1.001)	0.071
Food security				
Food secure	Ref	-	Ref	-
Marginal food insecurity	0.765 (0.393–1.492)	0.432	1.483 (0.529–4.154)	0.454
Moderate food insecurity	0.989 (0.625–1.566)	0.963	1.295 (0.643–2.611)	0.470
Severe food insecurity	0.661 (0.392–1.113)	0.119	0.467 (0.201–1.088)	0.078
Drink alcohol				
No	Ref	-	Ref	-
Yes	0.674 (0.501–0.906)	0.009	1.237 (0.632–2.420)	0.534
Smoke/vape				
No	Ref	-	Ref	-
Yes	0.566 (0.412–0.777)	<0.001	1.090 (0.516–2.302)	0.822
Diabetes knowledge (per %)	1.008 (0.999–1.018)	0.097	1.016 (0.999–1.033)	0.065
Waist circumference (per 1 cm)	1.010 (1.001–1.018)	0.024	1.016 (0.985–1.048)	0.327
Body fat percentage (per %)	1.035 (1.021–1.050)	<0.001	0.995 (0.947–1.045)	0.829
Body mass index (per 1 kg)	1.010 (0.995–1.025)	0.183	1.016 (0.949–1.089)	0.641
Blood pressure				
Normal	Ref	-	Ref	-
High blood pressure	2.434 (1.804–3.286)	<0.001	1.706 (0.862–3.377)	0.125

Note. COR = crude odds ratio; AOR = adjusted odds ratio; CI = confidence interval; Ref = reference category. *p*-values < 0.001 are presented as <0.001.
